# Correlation between human gut microbiome and diseases

**DOI:** 10.1016/j.imj.2022.08.004

**Published:** 2022-08-24

**Authors:** Barkha Madhogaria, Priyanka Bhowmik, Atreyee Kundu

**Affiliations:** a Department of Microbiology, Techno India University, West Bengal EM-4 Sector-V, Salt Lake City, Kolkata 700091, West Bengal, India; b Department of Biological Sciences, Adamas University, Barrackpore-Barasat Road, 24 Paragnas North, Jagannathpur, Kolkata, West Bengal, India

**Keywords:** Bacteria, Gut, Infections, Microbiome, Microbiota, Microflora

## Abstract

Human gut microbiome is a major source of human bacterial population and a significant contribution to both positive and harmful effects. Due to its involvement in a variety of interactions, gut microorganisms have a great impact on our health throughout our lives. The impact of gut microbial population is been studied intensively in last two decades. Extensive literature survey focusing developments in the field were searched in English language Electronic Databases like PubMed, Google Scholar, Pubag, Google books, and Research Gate were mostly used to understand the role of human gut mirobiome and its role in different human diseases. Gut microbiome in healthy subjects differs from those who suffer from diseases. Type 2 diabetes, obesity, non-alcoholic liver disease, and cardiometabolic diseases have all been linked to dysbiosis of the gut microbiota. Pathogenesis of many disorders is also linked to changes in gut microbiota. Other diseases like cancer, arithritis, autism, depression, anxiety, sleep disorder, HIV, hypertension, and gout are also related to gut microbiota dysbiosis. We focus in this review on recent studies looking into the link between gut microbiome dysbiosis and disease etiology. Research on how gut microbiota affects host metabolism has been changed in past decades from descriptive analyses to high throughput integrative omics data analysis such as metagenomics and metabolomics. Identification of molecular mechanisms behind reported associations is been carried out in human, animals, and cells for measure of host physiology and mechanics. Still many the mechanisms are not completely understood.

## Highlights

• Normal composition of gut microbiota, its diversity in number and type in different parts of the gut. Factors that play vital role in the development of gut microbiome.
• Role of gut microbiota in human body, which also explains that deviation in the composition can lead to different disorders.
• Deviation or dysbiosis with respect to different life threatening diseases like cancer, cardiovascular disease, bowel inflammatory disease and difficult-to-treat bacterial infections is explained.
• Future of the gut microbiota study and its role in treating diseases are discussed.

## Graphical abstract

**Figure:**
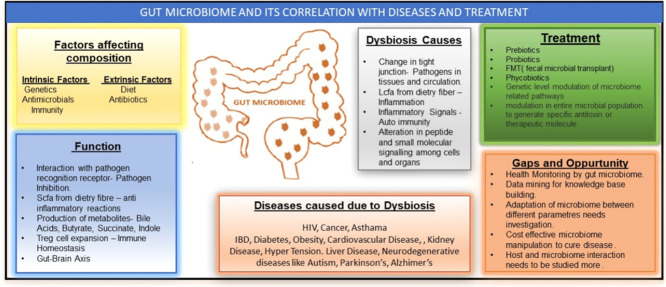

## 1 Introduction

There is far more microbial cell in human body than human cell. The process of colonization by microorganisms on human body's exposed surface such as skin, gut, mouth and vagina start immediately after birth. The aggregate of bacteria, fungi, and other microorganisms occurring on or within human forms microbiota and genes collectively that microbiota carries forms microbiome. The microbiota has beneficial and mutualistic association with their human host and has profound health and physiological impact. Most important and prominent component among human microbiota ecosystem is gut bacteria. The number and kind of bacteria are determined by physiological factors such as intestinal motility, pH, redox state, nutrition, host secretions (e.g., stomach acid, bile, digestive enzymes, and mucus), and the presence of an intact ileocecal valve in various parts of the gut. Aside from intrinsic characteristics, extrinsic factors such as antibiotic usage, disease, stress, aging, poor dietary habits, and lifestyle can all affect gut microbiota differences [1].

The special projects on human microbiome have established that immune environment change supposed to affect the gut flora which results in dysbiosis. “Diseases like cancer, cardiovascular disease, bowel inflammatory disease and difficult-to-treat bacterial infections due to antibiotic resistance have been linked with dysbiosis” [2].

Microorganisms and their metabolites play critical roles in human energy metabolism, nutrition absorption, immunological function, and other vital physiological functions. When commensalism between the host and the microbes is interrupted, a number of human diseases can result.

Many recent investigations have revealed specific strains of live microorganisms that, when administered in sufficient amounts, causes health advantages in the host. These are termed as probiotics. Scientists have also developed the concept of such food which promotes the growth of beneficial microbiota. These foods are called prebiotics.

In the past few years' vast technological improvements and diversity in knowledge have helped in the evolution of microbiome research, reinforcing our understanding of microbiome and its relationship with human health.

Therefore, the objective of this review is to analyze and summarize recent literature reports on the role of the gut microbiome, on human health and its effect on diseases.

## 2 Characterization of the gut microbiota: who is in there?

The deviation in healthy microbiota can be studied if we know which microorganisms resides in human gut in healthy state. According to an estimate microorganisms like bacteria, viruses, fungi, and protozoa that colonizes the gastrointestinal tract outnumbers human cell counting up to 100 trillion [3].

**Fig. 1:**
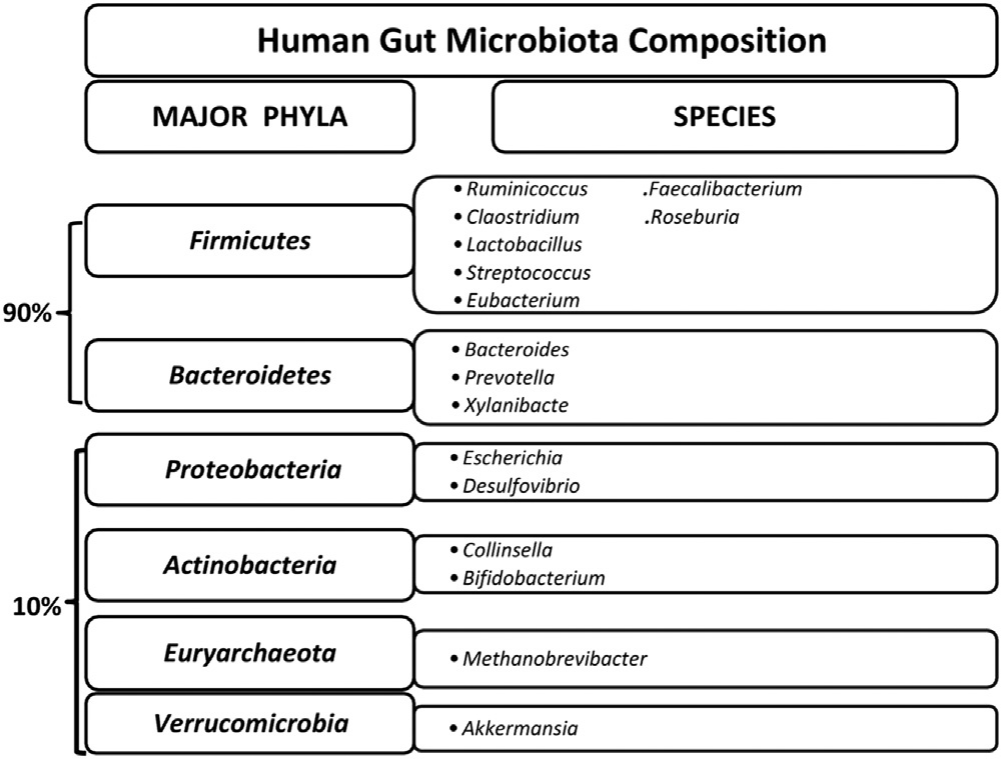
Six major phyla of human gut microbiota and their predominant species. (For interpretation of the references to colour in this figure legend, the reader is referred to the web version of this article.)

Due to vast difference in physiological conditions like pH which differs in different part of the gut, the bacterial and micro-organism's population also vary. 99% of the bacteria of entire gut microbiome are anaerobes. The inner environment of the gut has low oxygen levels which facilitate the expansion of strict anaerobic species of bacteria. In contrast cecum have high densities of aerobic bacteria. Gut is dominated with six major phyla- *Firmicutes* (e.g., Lactobacillus, *Ruminococcus, Clostridium, Eubacterium, Faecalibacterium, Roseburia*,Streptococcus species), *Bacteroidetes* (e.g., Bacteroides, Prevotella, Xylanibacte), *Proteobacteria* (e.g., *Escherichia, Desulfovibrio*), *Actinobacteria* (e.g., *Collinsella, Bifidobacterium*), *Euryarchaeota* (e.g., *Methanobrevibacter*), and *Verrucomicrobia* (e.g., *Akkermansia*) [4] (Fig. 1).

Survival rate of microorganisms is extremely low in stomach because of high acidity. By employing a small subunit of 16S rDNA for the first time in the gastric mucosa; identification of 1056 strains of non-*H. Pylori*, 127 phylotypes and 5 dominant genera of *Prevotella, Veillonella, Rothia, Fusobacterium,* and *Streptococcus* was done. Many recent studies used techniques like cloning, sequencing 16S rRNA and pyroseqencing is being used and identified that gastric microbiota contains primarily non-*H. pylori* and genera like *Neisseriae, Veillonella, Prevotella, Haemophilus, Porphyromonas, Rothia, Pasturellaceae Streptococcus, Propionibacterium*, and *Lactobacillus* were dominantly present [5].

Gram-positive cocci and bacteria that are rod shaped are predominantly present in small intestine. However, alkaline environment of the distant part of the small intestine promotes growth of *Enterobacteriacea* [4]*.* In Intestinal microbiota dominant phyla are: *Bacteroidetes, Firmicutes, Actinobacteria, Proteobacteria,* and *Verrucomicrobia*. Alone *Bacteroidetes* and *Firmicutes* constitute 90% of the whole population. *Veillonella, Eschericha, Bacteroids, Claustridium, Lactobacillus, Streptococcus* are also found in intestinal tract.

**Fig. 2:**
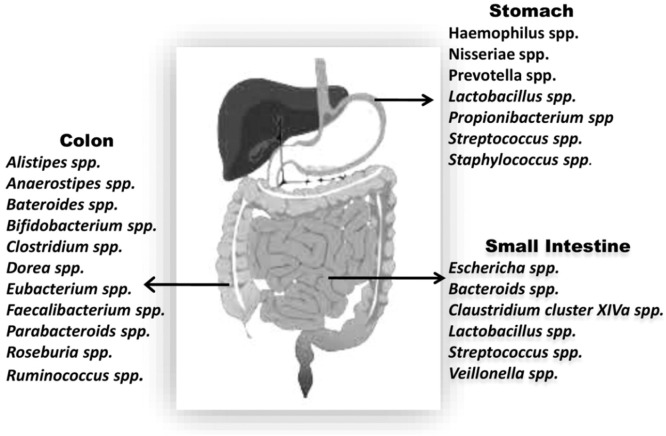
Dominant species in different parts of GI tract.

Due to significantly reduced enzyme activity, colon harbors vast microbial diversity. Bacterial population in colon are of Acidaminococcus, Faecalibacterium, Veillonella, Pseudomonas, Bacteroides, Bifidobacterium, Coprococcus, *Staphylococcus,* Enterobacter, Escherichia, Eubacterium, Fusobacterium, Klebsiella, Lactobacillus, Megamonas, *Salmonella*, Megasphaera, Peptostreptococcus, Enterococcus, Peptococcus, Proteus, Ruminococcus, and *Clostridium* species. Different species are in different numbers [5] (Fig. 2).

When transverse section of intestine was studied variation in composition of microbiota across the intestinal wall existed. For example, representing the luminal population were genera Bacteroides, Bifidobacterium, Streptococcus, Enterococcus, Clostridium, Lactobacillus, and Ruminococcus found in the feces, whereas epithelial crypts as well as mucus layer of the small intestine contains only Enterococcus, Clostridium, and Lactobacillus [6].

## 3 Establishment and evolution of the microbiota throughout life

While in uterus, babies are in amniotic fluid which is considered to be sterile traditionally, so babies are also considered sterile. Meconium which is the early feces sample of infant, it harbors negligible microbial and virus presence. The birth process has great impact in microbiota composition, babies those are delivered vaginally contain high concentration of *Lactobacilli* during the few initial days, because in vaginal flora Lactobacilli are present in higher number. Whereas delivery by C-section leads to presence of Clostridium species a facultative anaerobe. The gut microbiota composition throughout the first year of life is rather simple and *Actinobacteria* and *Proteobacteria* are the 2 phyla which primarily dominate the infant in their early stage of life [7].

By the last stage of first year of life, the convergence of bacterial microbiota into adult's starts, and by two and a half year of age microbiota resemblance is more to as of adult. One characteristic difference in microbiota of elderly with respective to young adult was founded that proportions of *Bacteroides* spp. and *Clostridium* groups are in greater number in elderly person gut microbiome [8].

The microbiota in centenarians exhibited some group-specific differences. Facultative anaerobes number such as *Escherichia coli* increases and alteration in number of butyrate producing bacteria eg, decrease in number of *Faecalibacterium prausnitzii* [9].

## 4 Factors affecting gut microflora

**Fig. 3:**
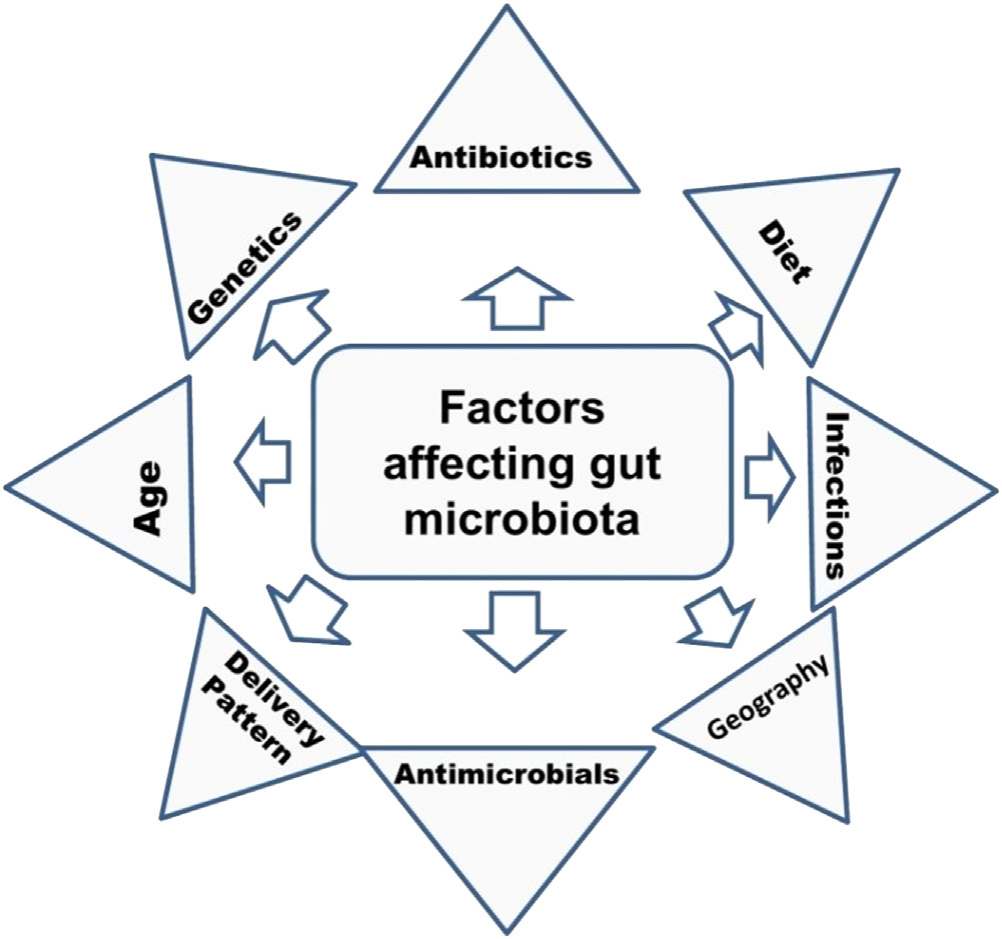
Factors influencing gut microbiota composition in human.

Many extrinsic and intrinsic factors affect composition of gut microbiota including antimicrobials, diet, adherence, and mucus and immunity of the host (Fig. 3).

### 4.1 Diet

Diet is the key factor in determining shape, structure and microbiota diversity of the gut. It is observed that in breast fed babies *Bifidobacterium* spp. is typically high, than in formula fed babies because *Bifidobacterium longum* utilizes fucosylated oligosaccharides which is present in mother's milk. Dominant species in vegetarians are Ruminococcus, Roseburia, and Eubacterium. These species are responsible for healthy, diverse gut microbiota that can metabolize insoluble carbohydrates [10]. In non-vegetarians decreased number of Firmicutes bacterial species and an increased Bacteroides spp. is a characteristic feature. According to several studies it was postulated that unlike vegetarian diet in which key feature is to promote fermentation of carbohydrate, western diet, involves amino acid fermentation, these results in short-chained fatty acid production as an energy resource which leads to production of harmful compounds. There is a correlation between diet and bacterial enetrotype. When diet with high content of animal fat was consumed *Bacteroides*-dominated enterotype was present and when carbohydrate-rich diet was consumed *Prevotella*-dominated enterotype was present.

Similarly, presence of *Bifidobacterium* spp. was in direct correlation with intake of dietary protein such as vegetable protein and fiber specifically soluble one which are found in green vegetables like beans, broccoli, asparagus, peas, and Brussels sprouts. Daily calorie intake criteria in human can be correlated to the presence of *Bacteriodes ovatus* population in gut. Presence of *Akkermansia* showed positive correlation with intake of saturated fats and negative correlation with respect to polyunsaturated fatty acids present in overall diet [11].

### 4.2 Antibiotics

Recent studies show persistent and rapid damage is caused by antibiotics to indigenous host-associated communities. These drugs amend gut microbiota to an extent of genomic, taxonomic, and functional capacity level. Some broad-spectrum antibiotics such as clindamycin which work against anaerobes have shown long-lasting effects on gut community composition.

The population of *Actinobacteria* is decreased by the use of Helicobacter pylori treatment. Similarly, *Ruminococcus* is decreased by ciprofloxacin use. In treatment of infection caused by *C. difficile* (CDI) vancomycin is used which is associated with decrease in number of gut microbes like Bacteroidetes, Fuminococcus, and Faecalibacterium and increases in Proteobacteria species [12].

In one of the studies, it was postulated that due to intake of antibiotics aromatic amino acids are released which acts as the mediators in dysbiosis of gut microbiota [13].

### 4.3 Genetics

Studies have shown that genetics influences number of specific bacteria in gut microflora. More similarity in microbiota is found in genetically related family members than in nonrelated members. In monozygotic twins' microbiotic similarity is more than in dizygotic twins [14].

### 4.4 Antimicrobials

Antimicrobial molecules are cationic peptides which interact with negatively charged bacterial membrane and destroy it. Therefore, restricts bacterial growth on mucosal surface. Gut species like *Firmicutes* and *Bacteroidetes* are resistant to these host-derived antimicrobial peptides. Bacterial species such as *B. fragilis* and Microaerophilic *Lactobacillaceae* expresses enzymes such as catalase, superoxide dismutase, and others to inactivate reactive oxygen species and thus can survive in gut [15].

More research is needed to understand and establish the mechanism of abovementioned factors leading to gut dysbiosis.

## 5 Functions of gut microbiota

### 5.1 Direct inhibition of pathogens

Through a barrier or competitive-exclusion effect, gut microbiota protects the body against infections. In addition, Microflora produces bacteriocins to inhibit the growth of their competitors.

### 5.2 Digestion

Carbohydrates which human are not able to digest are oligosaccharides, starches, fibers, and sugars like lactose.

Bacteria residing in the large intestine converts carbohydrates into short-chain fatty acids (SCFA). This process releases products like acetic, propionic and butyric acid. Having the ability to switch off the hunger signal from brain, propionate serves as satiety molecule and facilitates production of ATP in liver. Butyrate, on the other hand, induces apoptosis in malignant epithelial cells that line the large intestine, lowering the risk of bowel cancer while also providing energy to gut cells. Acetic acid is used by muscles, large number of gases like hydrogen, carbon dioxide, and odorless methane with small quantity of pungent odoriferous gases like hydrogen sulfide are by product of fermentation of dietary fibers carried out by gut [16].

### 5.3 Metabolism

Gut bacteria can create a range of critical vitamins for health and survival, as well as synthesize all essential and nonessential amino acids and perform bile biotransformation.

Many water-soluble vitamins, such as folic acid (B9), riboflavin (B2), biotin (B7), cobalamin (B12), nicotinic acid (B3), pantothenic acid (B5), and thiamine (B1), and fat-soluble vitamins, such as Vitamin K, can be synthesized by a microbial community rich in *Bifidobacterium*, Lactobacilli, and *E. coli*. It also aids in the absorption of nutrients such as magnesium, iron, and calcium [17].

### 5.4 Immune-system development

Gut bacteria ferment some food components and produce SCFA which induce rapid increase in the production of eosinophils, basophils, and neutrophils [18].

Specific receptors are present on the intestinal epithelium. These receptors identify and bind to specific bacteria-associated chemicals, causing the release of cytokines, protective peptides, and white blood cells as a result of the host immune response.

Some studies hypothesized that in early stages of life, stimulation and responsiveness of our immune system is controlled by intestinal microbiota, any alteration in healthy microbiota can lead to over reactive or inadequate responsive immune system in later life.

Among gut bacteria *Bacteroides fragilis* and several *Clostridia* species have been reported to cause an anti-inflammatory response, while some segmented filamentous bacteria have been found to cause inflammatory cytokine production. ‘Gut microflora helps to regulate antibodies production which further initiates B cells conversion IgA cells. IgA is an important antibody for mucosal gut environment. IgA helps in creating diversity in gut microflora and helps to get rid of inflammation causing bacteria thus maintains healthy gut bacteria and host environment [19].

### 5.5 The gut–brain axis

“Gut–Brain axis broadly includes the central nervous system, neuroendocrine and neuroimmune systems including the hypothalamic–pituitary–adrenal axis (HPA axis), sympathetic and parasympathetic arms of the autonomic nervous system including the enteric nervous system, the vagus nerve, and the gut microbiota” [19].

Many recent reviews have suggested that communication between the gut bacteria and the central nervous system influences the host's stress reactivity.

Recent studies have showed that many probiotic strains which are good gut bacteria have potential to be useful in the treatment of central nervous system disorders. Tested probiotics which have shown improvement in neural disorders contains bacteria of *Bifidobacterium* and *Lactobacillus* genera these have great potential to be useful for certain central nervous system disorders [20].

Obligate anaerobic bacterial population like *Bacteroides, Clostridium, Fusobacterium* etc. of our normal gut that are responsible for digesting complex dietry fibers into metabolites. Which are then absorbed by the host cells for different immunological purposes.

Some of the recent researches have proposed that colonocyte cell which is also called colonic epithelial cells helps host to maintain homeostasis in gut microbiota by consuming high levels of oxygen and maintains their oxidative metabolic stress, resulting in anaerobic condition in gut lumen maintaining gut homeostasis [21].

Changes in the anaerobic condition of the gut due to high fat diet, causes mitochondrial dysfunction by triggering production of hydrogen peroxide in the mitochondria [22].

Mitochondrial dysfunction also leads to reactive oxygen species formation which leads to dysbiosis but mechanism is unknown [23].

In case of antibiotics treatment it decreases the production of SCFA like butyrate, propionate, and acetate which increases inflammation. By impairing PPAR-γ signals in epithelial cells which in turn trigger synthesis of nitrogen oxide synthetase leading to formation of more nitrogen oxide which contributes in colonization of *Enterobacteriaceae* and creating dysbiosis. Modulation in regulatory T cells due to antibiotic treatment can also lead to imbalance of epithelial hypoxia resulting in dysbiosis [21].

Abrupt and sharp changes in the composition of healthy gut microbiota can involve multiple mechanisms and pathways and more research is needed to understand the mechanisms involved.

## 6 Gut microbiota and human diseases

The human microbiome is proven to be key component of human health. "Dysbiosis" is a state in which distinct changes happen in human microbiome. The impact is to such extant that microbiome has been proposed as "essential organ" of the human body [21].

There are enough studies to show that dysbiosis of the gut microbiota causes a wide range of diseases, including cardiovascular disease, gastrointestinal problems, allergies, obesity, and CNS-related diseases (Table 1) [22], [23], [24], [25], [26], [27], [28], [29], [30], [31], [32].

**Table 1:** Gut dysbiosis chart for different diseases.

Disease	Bacteria that decreases in number	Bacteria that increases in number	References
Colorectal cancer	*↓Prevotella, ↓Ruminococcus* spp.*, ↓Pseudobutyrivibrio ruminis*	*↑Acidaminobacter, ↑Phascolarctobacterium, ↑Citrobacter farmer,*	[60,61]
Colon cancer	*↓F*.prausnitzii,	*↑Akkermansia muciniphila*	
Gastric cancer	*↓Eubacterium* rectalie	*↑Clostridium,, ↑Fusobacterium,*	
Prostate cancer		*↑Lactobacillus ↑Firmicutes/Bacteroideted ratio*	
Obesity	*↓Bacteridetes ↓*Methanobrevibacter smithii	*↑Enterobacteria, ↑Ruminococcus gnavus*	[62]
IBD: Chron's disease	↓*Bacteroides,* ↓*Faecalibacterium prausnitzii* ↓*Bifidobacterium adolescentis*		[63]
Ulcerative cholitis	↓*Bifidobacteria*, ↓*Roseburia hominis* ↓*Faecalibacterium prausnitzii*, ↓*Lachnospiraceae,* ↓*Ruminococcaceae*		
Diabetes: Diabetes type1	↓*Lactobacillus,* ↓*Bifidobacterium,* ↓*Blautia coccoides,* ↓*Eubacterium rectal,* ↓*Prevotella,* ↓*Firmicutes*	↑*Clostridium,* ↑*Bacteroides,* ↑*Veillonella*	[64]
Diabetes type2	*↓Firmicutes, ↓Clostridia, ↓Lactobacillus, ↓Eubacterium rectale,*	*↑Bacteroids-Prevotella Verses Clostridiacocoides, ↑Betaproteo bacteria, ↑Bacteroidetes/Firmicutes ratio*	[65]
Cardiovascular disease		*↑*Clostridium, *↑*Lactobacillales, *↑Enterobacteriaceae* spp, *↑*Chryseomonas, *↑*Helicobacter, *↑*Firmicutes, *↑*Bacteroides	[66]
Liver disease	↓Alistipes, ↓Bilophila, ↓Veillonella, ↓Faecalibacterium, ↓Ruminococcus, ↓*Bifidobacterium*, ↓Prevotella, ↓Coprococcus*,* ↓Veillonellaceae, ↓Prevotella copri*,* ↓Faecalibacterium, ↓Haemophilus	*↑*Claustridum, *↑*Bacteroidetes, *↑*Betaproteobacteria, *↑*Lactobacillus spp.*, ↑*Collinsella*, ↑*Corynebacterium, *↑*Prevotellaceae, *↑*Ruminococcaceae, *↑*Sarcina, *↑*Sutterellaceae, *↑*Enterobacteriaceae, *↑*Bacteroidaceae	[67]
HIV	↓*Clostridia,* ↓*Bacteroidia,* ↓*Lactobacilli,* ↓*Bifidobacteria*	↑*Erysipelotrichaceae,* ↑*Proteobacteria,* ↑*Enterobacteriaceae,*↑*Candida albicans* ↑*Pseudomonas aeruginosa*	[68]
Autism	↓*Firmicutes,* ↓*Actinobacteria,* ↓*Actinobacteria*	↑*Bacteroides vulgates,* ↑*Desulfovibrio,* ↑*Proteobacteia*	[69]
Arthritis	↓*Bifidobacteria,* ↓*Bacteroides fragilis*		[70]

↓ Decrease in number ↑ Increases in number.

### 6.1 Cancer

Approximately 20% of all cancer cases in the globe are thought to be linked to the gut microbiota. Studies shows that F. *nucleatum* subdue host immune response and triggers cellular proliferation. Butyrate is important nutrient for healthy colon. Genera which produce Butyrate were almost absent in colorectal cancer (CRC) patient's stool. When some cancer samples were studied, *Prevotella* species were not present at all. Whereas, bacteria like *Acidaminobacter, Phascolarctobacterium*, and *Citrobacter* were present in significant number in CRC patient's stool samples. Whole grains and rich fiber having diet can lower the occurrence of CRC related to positive presence of *F. nucleatum. Bifidobacterium longum* indicated strong antitumor activity in colon cancer treatment [33].

*H. pylori* is related to major risk of stomach cancer in humans. New researches suggested that elevated population of bacterial spp. like *Fusobacterium* and *Clostridium* can be observed in stomach cancer patients. For the diagnosis of gastric cancer bacterial strains are used as markers eg, *C. colicanis* and *F. nucleatum*. Prostate cancer is highly influenced by *Bacteroides* massiliensis [34].

In case of cancer, due to disturbance in intestinal barrier bacteria from intestinal lumen reaches in the tissue which triggers many inflammatory responses one of them is induction of pattern recognition receptors which are linked to cancer progression these receptors include mainly membrane bound TLRs (Toll-like receptors) and NLRs (Nod-like receptors).

Many researches have been done and 2 main hypotheses are there to suggest the link between dysbiosis and cancer. One of them is alpha drug hypothesis in which researchers proposed that presence enterotoxigenic bacterial species *Bacteroides fragilis* disrupts the colonal microbiota causing inflammatory responses including cytokines like IL-17, TNF-α and TH17 etc. which promotes cancer progression.

In another hypothesis model named bacterial driver-passenger driver is referred to bacteria Bacteroides fragilis causes inflammation due to this inflammation response genotoxins like CDT, BNF, BST etc. are produced this causes cell proliferation and some serious mutations leading to adenoma formation. Now another bacterial species Fusobacterium spp. Works as passenger bacteria causing the progression of that adenoma formed [35].

### 6.2 Inflammatory bowel disease

Irritable bowel illness is a type of inflammatory bowel disease (IBD). Ulcerative colitis (UC) affects only the colon. *Lactobacilli* species were found to be less in number during active phase of disease but some species such as *Lactobacillus salivarus, Lactobacillus manihotivorans*, and *Pediococcus acidilactici* were detected during remission phase and not during active inflammation. Studies suggested that pathology and development of colitis is related to higher *E. coli* load. *Roseburia hominis* and *Faecalibacterium prausnitzii* were present in reduced amount in fecal samples of UC patients. Bacterial species like *Lachnospiraceae* and *Ruminococcaceae* were also present in fewer amounts [36].

Crohn's disease (CD) patients have a greater number of *Enterobacteriaceae* than in healthy individual. Other investigations have discovered a drop in the number of *Dialister invisus*, an unidentified *Clostridium* cluster XIVa species, *Faecalibacterium prausnitzii*, and *Bifidobacterium adolescentis*, as well as an increase in the number of *Ruminococcus gnavus*. Increased numbers of *Enterobacteriaceae, Pasteurellaceae, Veillonellaceae,* and *Fusobacteriaceae* bacteria but lower numbers of *Erysipelotrichales, Bacteroidales,* and *Clostridiales* bacteria was identified in pediatric CD patients [37].This population identification can be used as pathological identification of this disease and structuring probiotic for the treatment can also be done.

Several animal studies have taken place to establish the correlation between dysbiosis and IBD (inflammatory bowel syndrome) and CD (Chron's Disease) but it is hard to postulate that dysbiosis leads to IBD or Chron's disease. Rather gut microbiota has limited the role in pathogenesis of diseases, instead it is a marker. Dysbiosis develop later in IBD patients and can lead to progression and chronicity of the disease. Gut microbiome contributes to modulate intestinal inflammation and certain immune responses. SCFA is a metabolite that maintains mucosal integrity by epithelial repair pathway, inflammasome and by production of cytokine IL-18. Another metabolite is bile acids (BAs) which help to induce anti-inflammatory process against Cholitis. Indole is also a metabolite which is produced by gut microbiota utilizing tryptophan. It regulates mucosal immunity with the help of receptors like polycyclic aromatic hydrocarbon receptors. So, in case of IBD and CD dysbiosis happens which modulates above mentioned activity in the gut leading to pathogenicity of these diseases. [38,39].

### 6.3 Metabolic disorders

#### 6.3.1 Obesity

Gut microbial composition is highly influenced by our dietary habits. Due to a high fat diet, the gut microbiome is altered, with higher amounts of *Firmicutes* and *Proteobacteria* and decreased levels of *Bacteroidetes*.The ratio of *Firmicutes* to *Bacteroides* has been linked to body weight, with obese people having a larger ratio. Obesity can also be caused by *Clostridium difficile* infections. Obesity is affected by chronic inflammatory status induced by gut bacteria or metabolites that regulate the microbiota-brain-gut axis by its metabolites [36].

Conjugated linoleic acid (CLA) is an important fatty acid that aids in the prevention of obesity. Four *Bifidobacterium breve* strains, one *Bifidobacterium bifidum* strain, and one *Bifidobacterium pseudolongum* strain were able to synthesize various CLA and conjugated-linolenic acid isomers from dietary supplements. *Bacteroides* spp., which is found in the gut microbiome, has been reported to play a role in obesity prevention.

For morbid obesity, bariatric surgery is a popular treatment option. Weight loss after bariatric surgery is also associated with increase in *B. thetaiotaomicron* abundance and decrease serum glutamate levels [40].

#### 6.3.2 Liver disease

Beneficial substances produced by liver are absorbed by gut. Intestinal microflora produces ammonia, ethanol and acetaldehyde; liver metabolizes these products and controls cytokine production and Kupffer cell activity. The severity of Concanavalin-A (ConA)-induced hepatitis is exacerbated when gut flora is suppressed by antibiotics. Intestinal bacteria that produce hydrogen have been shown to reduce ConA-induced inflammation in studies [41].

Liver damage due to high alcohol intake is also associated with dysbiosis of gut microbiota. Endotoxins and luminal bacterial metabolites or products may be the cause for alcohol related liver damage and can triggers alcohol-induced endotoxemia. One of the dreaded complications of liver disease Hepatic Encephalopathy (HE) is a common liver disease. One critical factor for the pathogenesis of HE is production of ammonia from amino acids through de-amination by some urease-positive bacteria. HE when treated with probiotics showed effectiveness compared to prebiotic and antibiotics treatment [41].

#### 6.3.3 Diabetes

Diabetes is a metabolic disease which is strongly associated with gut microbiome. There are 2 types of diabetes type-1 diabetes (T1D) and type-2 (T2D) are major concern of the world.

Studies showed that in children with T1D have increased abundance of *Veillonella, Clostridium*, and *Bacteroides* and decreased abundance of *Lactobacillus, Eubacterium rectale, Blautia coccoides*, and *Bifidobacterium* group. Furthermore, negative correlation was established between plasma glucose level and *Bifidobacterium, Lactobacillus* spp. and *Firmicutes, Bacteroidetes* spp., and positive correlation between clostridium and plasma glucose level. The phylum *Firmicutes* was also reduced specially *F. prausnitzii* species while *B. fragilis* was present in lower amount. The ratios of *Bacteroidetes* to *Firmicutes* were found to have a positive connection with plasma glucose levels. *Lactobacillus* genus was also in lower abundance in T2D patients and Bifidobacterium was in higher abundance. Presence of class *Betaproteobacteria* was also positively correlated to plasma glucose level [42].

For the fact *A. muciniphila* found to be related to improved mucous production and delayed diabetes development. Therefore, it can be used as probiotic treatment for T1D. Future antidiabetic drugs will target specific bacterial strains that cause an imbalance of amino acid metabolism [42].

Dysbiosis leads to many metabolic disorder in and is linked directly to obesity and fatty liver disease and is a factor responsible for the occurrence of diabetes mellitus.

Alteration in normal gut microbiota directly affects the liver vascular barrier causing permeability to bacteria and other derived metabolites which triggers the immune system to generate inflammation leading to pathogenesis of MFLD. This can also affect the insulin tolerance factor inducing diabetes mellitus [43].

Indigestible carbohydrates and used by gut microbiome to produce SCFA and succinate these metabolites related to satiety and energy expenditure and plays important role in the obesity. SCFAs are also directly linked in increasing energy consumption and lipid oxidation. This can prevent obesity by upregulating expression of some heat generation and lipid oxidation related proteins like PPARγ, PGC_1α_, UPC_1_, CPT-1, and UCP_2_. Researches have shown that succinate enhances intestinal gluconeogenesis which in turn triggers Gut-Brain glucose signaling by binding to the receptor name GPR_91_ [39].

### 6.4 Cardiovascular diseases

Cardiovascular illnesses, such as atherosclerosis (acute coronary syndrome and stroke), heart failure, and hypertension. Atherosclerotic cardiovascular disease is characterized by high presence of *Enterobacteriaceae* and *Streptococcus* spp. in gut microbiome [40].

"In atherosclerotic plaques phylum *Proteobacteria* (Chryseomonas and Helicobacter genera) and phylum *Firmicutes* (Anaeroglobus, Clostridium, Eubacterium, Lactobacillales, and Roseburia genera) is found in abundance in gut cavity. Other bacterias like Enterobacteriaceae, Streptococcus spp, Lactobacillales, and Collinsella population is altered in the gut among cardiovascular patients". In fact, Bacteroides, Clostridium and Lactobacillales are considered to be diagnostic markers in patients suffering from coronary artery disease. Usage of subsp. lactis LKM512 of *Bifidobacterium animalis* as probiotic has shown reduced TMA levels and certain bacterial group such as (*Clostridia, Clostridiales*, and *Lachnospiraceae*) which produces TMA, thus reducing the risk of arteriosclerosis [44].

Gut dysbiosis leads to alteration of reverse the cholesterol transport system causing a condition called metabolic endotoxemia. Dysbiosis leads to intestinal permeability leading to increase the level of LPS in blood circulation which triggers the expression of cytokines and cell adhesion molecules.Which in turn induces monocytes adhesion to endothelial layer causing atherosclerosis progression, promotes inflammation and formation of foam cells to remove excess LPS from the blood.

Butyrate, Trimethyl-amine N oxide, and bile acid are metabolites of gut microbiota. Gut microbiota helps to regulate the atherosclerosis progression by modulating bile salt hydrolase activity. Higher bile salt hydrolase higher the bile acid formation and lower the LPS.

Trimethyl-amine N oxide levels are directly linked to cholesterol catabolism and modulation of reverse cholesterol transport. Impairment of RCT and cholesterol catabolism due to dysbiosis can lead to cardiovascular disease risk [45].

Dysbiosis modulates butyrate production which can lead to anti-inflammatory impairment causing monocyte adhesion and plaque development causing atherosclerosis increasing cardiovascular disease risk.

#### 6.4.1 Hypertension

SCFAa chemical generated by certain gut bacteria, lowers blood pressure by binding to the olfactory receptors gpr41, gpr43, and olfO79 found in the kidney, heart, sympathetic ganglia, and blood vessels. By product of gut microbiome, hydrogen sulfate, has direct effect on blood vessels and regulates blood pressure. SCFA also reduce inflammation and reduce sympathetic nerve activity by directly mediating immune cell response [46].

### 6.5 HIV

Dysbiosis plays an important role in HIV infection pathogenesis. The vital site for early HIV replication is CD4^+^ T cells, human GI tract is a reservoir for these types of cells hence making GI tract a reservoir for HIV. Chronic systematic inflammation, translocation of immune stimulatory microbial products and disturbance of the intestinal immune barrier results in AIDS from HIV infection [47].

Wolf B.W. found that in HIV infected patients 579 taxa were present in higher number and 45 taxa were present in lower number. Viremic untreated HIV-infected patients also called as VU subjects were compared to HIV− subject samples and it was observed that *Erysipelotrichaceae* in the class *Mollicutes* which is responsible for obesity and Cardiovascular morbidity is the mostly found in VU subjects and pro-inflammatory pathbionts from *Enterobacteriaceae* family including *Shigella, Salmonella, Escherichia, Serratia* and *Klebsiella* species are enriched genera in such patients. In the mucosae of Vu patients strains of *Pseudomaonas, Campylobacter* spp*.,* and *Pseudomonas* were found to be high in number. These opportunistic pathogens are source of bacteremia in HIV patients.

In HIV patients, it was found that *lactobacilli* and *Bifidobacterium* from gut bacterial population declined to great extent and pathogenic species like *Candida albicans* and *Pseudomonas aeruginosa* were higher in number [48].

#### 6.5.1 Autism and other neurological disorder

Many experimental studies postulated that autistic patients not only show dysbiosis but also alterations in metabolites production.

Some strains of other gut bacteria such as *Faecalibacterium* and *Ruminococcaceae* are related to the expression of zonulin which is a protein which is responsible for modulating gut permeability. Increased level of zonulin is linked to behavioral disregularity [49]. SCFA is found to be lower in autistic patients who are correlated with presence of *Faecalibacterium, Ruminococcus*, and *Bifidobacterium* species. *Clostridia* species are found in abundance in ASD patients which is responsible for the production of propionate [50].

"In the urinary and fecal sample of ASD patient's p-cresol compound was present in higher amount and bacteria such as *Clostridium scatologenes, Lactobacillus*, and *Pseudomonas* are responsible for the conversion of toluene to p-cresol". P-cresol is associated with gut dysfunction and worsening of ASD symptoms. Results were attained related to ratio of *Firmicutes* to *Bacteroidete* and it was established that the ratio usually increases.

Whereas, *Alistipes, Bilophila, Veillonella****,*** *Faecalibacterium, Ruminococcus, Bifidobacterium* and Genera *Prevotella, Coprococcus,* unclassified *Veillonellaceae, Prevotella copri, Faecalibacterium prausnitzii,* and *Haemophilus parainfluenzae* were detected in lower abundance in ASD patients [50].

In case of nervous system disorder gut-brain-axis plays a pivotal role in brain functioning as well as development of neurons. Brain functioning is modulated by neurotransmitters like GABA and 5-HT. Which is modulated by the action of different types of microbial metabolites like SCFA, BA, etc. These metabolites a well as some gut flora also act on vagus nerve and intestinal nervous system affecting brain functioning and behavioral conduct. On the inflammatory level there is a contribution of dysbiosis on Microglia and systemic cytokines.

Many researches have shown that gut microbiota is related to the pathogenicity of Alzheimer's disease. By modulating β-amyloid protein pathology, microbial infection can lead to its accumulation causing nerve damage and severity.

Similarly, in case of Parkinson's disease α-synuclein is accumulated in the nerves. Microglia's activity is increased because of dysbiosis. This confirms the role of dysbiosis in this disease [39].

#### 6.5.2 Arthritis

Rheumatoid arthritis (RA) disease is explained as a systemic and chronic inflammatory human disease. The breakdown of joint cartilage and bone as a result of a persistent immune response is one of the most common symptoms. Commensal bacteria influence immunological responses by affecting dendritic cell development and function, as well as T cell subsets [47]. Disproportion of ratio of Th17/Treg cell and Toll-like receptors of antigen-presenting cells is influenced by dysbiosis in gut microbiome. *Bacteroides, Akkermansia* or the anti-inflammatory *Faecalobacterium prausnitzii* bacteria were depleted and *Prevotella* and *Ruminococcus* were in abundance in RA patients leading to inflammation [51].

Metagenomic shotgun techniques carried out in various studies suggested that RA patient's shows significant difference in gut microbiome composition than in healthy person. Presence of *Actinobacteria* triggers pathogenesis In RA patients. *Collinsella* sp. And *Eggerthella* sp. can be used as markers. Zhang et al. studied the fecal and saliva sample of RA patients and found out lower presence of *Haemophilus* sp. and higher presence of *Lactobacillus salivarius*. It was found out that RF –positive patients were mostly colonized by phylogenetic group D and RF-negative patients had *E. coli*, phylogenetic group B2 type [52].

### 6.6 Other diseases

#### 6.6.1 Asthma

The increased prevalence of asthma is associated with a set of 2 hypotheses. One is hygiene hypothesis and second one is western pattern diet. Western diet pattern includes mostly simple sugars and lacks whole grain and fiber. SCFA, a microbial metabolite byproduct production is highly dependent on gut microflora, and diet containing grains and fibers, which is a key trigger point for immunomodulation in humans. SCFA controls immune signaling to prevent asthma, SCFA levels that are lower are linked to the condition. Lower SCFA levels have been linked to bacterial species such as *Veillonella, Lachnospira, Faecalibacterium, Rothia* and *Faecalibacterium* [53].

#### 6.6.2 Gout

Gout is an autoinflammatory disease related to elevation in blood uric levels result of purine metabolism disorder. High abundance of uric acid in blood leads to its crystallization and accumulation of uric acid crystals in joints leading to acute pain. Gout patients when examined showed presence of *Bacteroides caccae* and *Bacteroides xylanisolvens*. Serious inflammatory responses were triggered due to the presence of *B. caccae* in gout patients. Bacteria *Faecalibacterium prausnitzii* and *Bifidobacterium pseudocatenulatum* which have anti-inflammatory properties were found to be depleted which also affects butyrate production necessary for gut health [53].

#### 6.6.3 Kidney diseases

Many studies have shown that intestinal bacteria are involved in kidney diseases. In patients suffering with chronic kidney disease; a metabolite p-Cresyl (p-CS) circulates in the blood and accumulates in the blood of kidney patient also known as uremic retention solute. Intestinal microbial composition plays an important role in production of p-cresol making it an identification marker for the disease [53].

## 7 Precision medicine and gut microbiome

Customized therapies for individuals or precision medicine are the future of human disease treatment. Gut microbiome or the second genome of the human body has an obvious effect on the precision medicine therapy designing. Microbiome can modulate the pathology of a disease by altering the immune response to it [54]. For example, differences in the number of the bacteria A. muciniphila correlated with the response of the cancer patients to PD-1/PD-L1 blockers [55,56]. Studies on other drugs like berberine, NSAIDs, histamine-2 blockers also confirm that gut microbiota influences the variability of drug responsiveness [57,58]. Selective restriction of gut Enterobacteriaceae using tungstate led the gut to return to its normal state of microbiome status and resulting in the reduction of colitis induced inflammation [59]. Gut microbiota-drug interaction: modulation of the metabolism of drugs. Gut microbes synthesizes array of different enzymes which can metabolize different drugs.

Fecal microbiota transplant (FMT) is a method where stool from a healthy donor is placed in the gastrointestinal tract of an individual to directly influence and normalize the gut microbiome composition [60]. It has been approved for the treatment of recurrent Clostridium difficile infection (CDI). Various studies have demonstrated the efficiency of FMT in the treatment of CDI [61,62]. Sky rocketing the success rate to about 90% while antimicrobial therapy success rate is quite less which is about 20%–30% [63], [64], [65]. Many clinical trials are shown to be effective against irritable bowel syndrome as well. In a systemic review published in 2021 reported success rates for IBD like ulcerative colitis, Chron's disease and pouchitis and their rate of remission is 36%, 50.5%, 21.5%, respectively [66], [67], [68]. Case reports and animal models demonstrated the effectiveness of FMT against an array of different disorders like metabolic disorders, insulin sensitivity, multiple sclerosis, autism etc. [69,70]. Clinical trials done in 3 groups with all male patients in 2018 for FMT to treat obesity and metabolic disorder was done. After 6 weeks of FMT treatment insulin sensitivity was increased in some patient's hemoglobin A1c was increased. But other criteria like HDL, LDL, cholestrol, and BMI showed no changes [71]. Melanoma immunotherapy had also been shown deliver positive effects with FMT in animal model and clinical trials [72]. FMT has also shown to relieve the neurological problems after traumatic brain injury [73].

This therapeutic strategy is very personalized and has individualized effect. Their success depends on factor such as host immunity. There is no such drawback of these strategies but lack of human trials studies. Moreover, gut microbiome engineering can be done to produce personalized advance therapeutic medicine.

## 8 Discussion

We live with large number of microorganisms in our gut. We have progressed in the analysis of composition of gut microbiome and key metabolites produced. Gut microbiome study can play a big role in disease diagnosis through stool, health screening, and monitoring. Use of prebiotic, probiotic, and fecal matter transplant can be used by health practitioners to treat chronic diseases like diabetes, IBD, obesity, etc. Drugs that can target alteration of gut microbiome composition can be a promising therapy. Gut microbiota studies can include health intervention by manipulating microbiome such as the mechanism and effect for probiotic and symbiotic have not been studied well, fecal microbiome transplant standards are not yet established, reproducibility of gut microbiome is a challenge. Structuring of community of microbiome is needed to be studied more efficiently to postulate which part of microbiome was critical to the development of disease. Interaction between host and microbiome is not well addressed yet. The adaptation of microbiome between different parameters should be investigated.

## 9 Conclusion

We have emphasized all potential factors which are relevant for the composition and abundance of bacterial species in gut in this review. This analysis excludes several less-studied factors that may play a role in gut microbiota growth, such as "maternal lifestyle (urban or rural) and fetal swallowing of amniotic fluid" [53]. This review also discusses the biogeographic stratification of microbiota in the gut. A close symbiotic link exists between the gut bacteria and the host. Disruption of the natural microbiota composition causes a variety of diseases, including gastrointestinal, metabolic, immunological regulation, and neurodevelopmental issues. For overall health, gut microbial homeostasis should be maintained. Many microbial metabolites have been discovered to play a function in decreasing spread and inducing apoptosis in human cancers. As a result, more research is needed to discover these metabolite-producing gut bacteria for therapeutic purposes. The impact of prebiotic and probiotic use on human health should be explored further in order to develop better medicines and diagnoses.

The metabolomics methodologies have substantially enriched our understanding of the relationship between gut microbiota and composition, as well as its impact on health and disease. The data collected from large-scale sequencing projects such as the Human Microbiome Project and the Earth Microbiome Project can be utilized to improve and translate our knowledge of the microbiome and enhance our health. Data that are generated through these enormous projects can be used to enhance and translate our understanding of the gut microbiome. Which can further help health practitioners to modulate gut microbiota to maintain and enhance human health. Human genome sequencing can be utilized to improve the prevention and treatment of many chronic diseases, including cancer. More research into the genetic sequencing of gut microbiota will change our therapy approach. A better understanding of the microbiota will aid in the reduction of negative events, as well as the expense of healthcare and treatment techniques.

## 10 Future perspective

We live with large number of microorganisms in our gut. We have progressed in the analysis of composition of gut microbiome and key metabolites produced. Gut microbiome study can play a big role in disease diagnosis through stool, health screening and monitoring. Use of prebiotic, probiotic and fecal matter transplant can be used by health practitioners to treat chronic diseases like diabetes, IBD, obesity etc. Drugs that can target alteration of gut microbiome composition can be a promising therapy. Gut microbiota studies can include health intervention by manipulating microbiome such as the mechanism and effect for probiotic and symbiotic have not been studied well, fecal microbiome transplant standards are not yet established, reproducibility of gut microbiome is a challenge. Structuring of community of microbiome is needed to be studied more efficiently to postulate which part of microbiome was critical to the development of disease. Interaction between host and microbiome is not well addressed yet. The adaptation of microbiome between different parameters should be investigated.

## Funding

This research did not receive any specific grant from funding agencies in the public, commercial, or not-for-profit sectors.

## Acknowledgments

The authors thank Techno India University, West Bengal, for support and encouragement during this study.

## Declaration of competing interest

The authors have declared no conflict of interest.

## Data available statement

No new data or model was used in this research.

## Ethics statement

Ethics approval were waived for this study because no patients' data were reported.

## Informed consent

Infromed consent was waived for this study because no patients' data were reported.
